# The descriptive epidemiology of total physical activity, muscle-strengthening exercises and sedentary behaviour among Australian adults – results from the National Nutrition and Physical Activity Survey

**DOI:** 10.1186/s12889-016-2736-3

**Published:** 2016-01-25

**Authors:** Jason A. Bennie, Zeljko Pedisic, Jannique G. Z. van Uffelen, Joanne Gale, Lauren K. Banting, Ineke Vergeer, Emmanuel Stamatakis, Adrian E. Bauman, Stuart J. H. Biddle

**Affiliations:** 1 Active Living and Public Health Program, Institute of Sport, Exercise and Active Living (ISEAL), Victoria University, Melbourne, VIC Australia; 2 Prevention Research Collaboration, Sydney School of Public Health, University of Sydney, Sydney, NSW Australia; 3 Charles Perkins Centre, University of Sydney, Sydney, NSW Australia; 4 Exercise and Sport Sciences, University of Sydney, Sydney, NSW Australia

**Keywords:** Public health surveillance, Strength training, Physical activity, Sitting

## Abstract

**Background:**

The current Australia's Physical Activity and Sedentary Behaviour Guidelines recommend that adults engage in regular moderate-to-vigorous-intensity physical activity (MVPA) and strength training (ST), and minimise time spent in sedentary behaviours (SB). However, evidence about the specific individual and concurrent distribution of these behaviours in Australia is scarce. Therefore, the aim of this study was to determine the prevalence and sociodemographic correlates of MVPA, ST and SB in a national-representative sample of Australian adults.

**Methods:**

Data were collected using face-to-face interviews, as part of the National Nutrition and Physical Activity Survey 2011–12. The population-weighted proportions meeting the MVPA (≥150 min/week), ST (≥2 sessions/week) and combined MVPA-ST guidelines, and proportions classified as having ‘low levels of SB’ (<480 min/day) were calculated, and their associations with selected sociodemographic and health-related variables were assessed using multiple logistic regression analyses. This was also done for those at potentially ‘high-risk’, defined as insufficient MVPA-ST and ‘high-sedentary’ behaviour.

**Results:**

Out of 9345 participants (response rate = 77.0 %), aged 18–85 years, 52.6 % (95 % CI: 51.2 %–54.0 %), 18.6 % (95 % CI: 17.5 %–19.7 %) and 15.0 % (95 % CI: 13.9 %–16.1 %) met the MVPA, ST and combined MVPA-ST guidelines, respectively. Female gender, older age, low/medium education, poorer self-rated health, being classified as underweight or obese, and being a current smoker were independently associated with lower odds of meeting the MVPA, ST and combined MVPA-ST guidelines. A total of 78.9 % (95 % CI: 77.9 %–80.0 %) were classified as having low levels of SB. Females, older adults and those with lower education were more likely to report lower levels of SB, whilst those with poor self-rated health and obese individuals were less likely to report lower levels of SB (i.e. SB = ≥480 min/day). A total of 8.9 % (95 % CI: 8.1 %–9.6 %) were categorised as individuals at potentially ‘high-risk’. Those with poorer self-rated health, obese individuals, those aged 25–44, and current smokers were more likely to be in the ‘high risk’ group.

**Conclusions:**

The large majority of Australian adults do not meet the full physical activity guidelines and/or report excessive SB. Our results call for public health interventions to reduce physical inactivity and SB in Australia, particularly among the subgroups at the highest risk of these unhealthy behaviours.

## Background

Non-communicable diseases (NCDs) are responsible for 68 % of all deaths worldwide [1]. Physical inactivity is among the leading preventable causes of NCDs [2]. It is estimated to cause 6–10 % of deaths related to coronary heart disease, diabetes, and breast and colon cancer [3]. It is the fourth ranked mortality risk factor; after hypertension, tobacco use and high blood glucose [3, 4], and it causes 9 % of premature mortality [3]. Estimates based on self-report data suggest that globally 40 %–60 % of adults are insufficiently active for health [5]. Consequently, reducing rates of physical inactivity has been described as a key 21^st^ century public health challenge [2, 6, 7].

Until recently, physical activity recommendations were primarily based around the accumulation of moderate-to-vigorous-intensity aerobic physical activity (MVPA) (e.g. walking or jogging) [8, 9]. However, evidence from epidemiological studies and controlled trials suggests that regular participation in muscle-strengthening activities (e.g. weight or resistance training) may provide additional benefits for musculoskeletal (e.g. reducing the risk of sarcopenia, osteoporosis and osteoarthritis) [10], metabolic (e.g. reducing the risk of metabolic syndrome and type 2 diabetes) [11, 12] and mental health (e.g. reducing the symptoms of anxiety) [10]. Moreover, recent studies have shown that, when compared to one activity mode alone, there may be greater metabolic health benefits of combining MVPA and muscle-strengthening activities [12–15].

In addition, sedentary behaviour (excessive sitting as opposed to insufficient physical activity) has recently emerged as a potential independent risk factor for poor health [16]. Prospective studies have shown that adults who accrue high volumes of sedentary behaviour are at a higher risk of developing chronic diseases such as type 2 diabetes [17–19] and cardiovascular disease [20], as well as at a higher risk of all-cause and disease-specific mortality [21–25]. Importantly, the association between prolonged sitting and detrimental health outcomes remains significant even after adjustment for time spent in MVPA [16, 26–28], indicating that, for optimal health benefits, people should both be active and limit their time spent in sedentary behaviour.

For the prevention and management of chronic diseases, leading global and national health authorities, such as the World Health Organization (WHO), the U.S. Department of Health and Human Services and the Australian Government, Department of Health, recommend that adults should participate in: (i) at least 150 min/week of moderate (e.g. brisk walking) or 75 min/week of vigorous-intensity physical activity (e.g. jogging), or an equivalent combination of both, and (ii) 2 or more days per week of muscle-strengthening activities involving major muscle groups [7, 29–31]. Additionally, authorities within Australia and the U.S. recommend that adults limit time spent in sedentary behaviours [29, 30]. At present there is no consensus on the threshold relationship between sedentary behaviour and poor health [16]. Hence, current sedentary behaviour reduction guidelines typically state that adults should ‘limit prolonged periods of time spent in sedentary behaviours’, and ‘frequently break up long periods of sitting’ [29, 30, 32].

Previous population-based studies have mostly focused on assessing the prevalence and correlates of MVPA [33–35]. Only few studies examined the proportion of people meeting the recommendations for strength training in national-representative samples.

Estimations range from 21.9 to 31.7 % within the U.S and UK [36–40] and from 9.4 to 15.5 % in Australia [41–43]. Data about meeting the combined MVPA-muscle-strengthening guidelines are even scarcer. Previous studies have shown that 18.2 to 20.6 % of U.S. adults meet both MVPA and strength training guidelines [36, 44, 45], but no such estimates exist for the Australian population. Furthermore, international studies have reported that adults from different countries sit on average between 135 and 360 min/day, whilst the prevalence rates of total sitting time above 9 h/day ranged between 2.6 and 34.9 % [46, 47]. No previous international or national studies have concurrently assessed the prevalence and correlates of MVPA, muscle-strengthening activities and sedentary behaviours, and clusters of these behaviours in a representative sample. Such data are essential to inform comprehensive interventions aimed to reduce physical inactivity and prolonged sitting, and to identify populations at the highest risk.

The aim of this study was, therefore, to determine the prevalence of adherence to the public health MVPA and muscle-strengthening activity guidelines, and high level of sedentarism among a population-representative sample of Australian adults and to investigate their sociodemographic and health-related correlates.

## Methods

This study accessed the data from the Australian Bureau of Statistics (ABS), National Nutrition and Physical Activity Survey (NNPAS) 2011–12. The NNPAS is a sub-component of the larger ABS Australian Health Survey (AHS), 2011–13, which is a national survey designed to provide detailed information on the health and wellbeing of the Australian population. Data from the NNPAS 2011–12 are are publicly accessible. The NNPAS 2011–12 was conducted between May 2011 and June 2012, and used a stratified multistage area sampling of private dwellings to ensure recruitment of a representative sample of Australian adults.

Detailed information on the survey design, data collection, and response rates can be found elsewhere [48]. The Australian Bureau of Statistics Research Ethics Committee approved all study protocols and written informed consent from all participants was obtained [48]. Upon given consent, data were obtained by face-to-face interviews in respondents’ homes by trained interviewers using Computer Assisted Personal Interview (CAPI) system.

The initial sample size (14,363 households) was reduced to 12,366 dwellings after sample loss in the field stage. Of the remaining dwellings, 9519 (77.0 %) fully or adequately responded to the first interview [48]. A total of 12,153 participants (aged 2–85 years) fully or adequately completed the survey. For the present study, we used data from adults aged 18–85 years, leaving a final sample of 9435 respondents. Each of the respondents was given a selection probability weight, which is reflective of how many people in the population they represent. More information on weighting in the NNPAS can be found on the ABS website [48].

### Measures and data management

The questionnaire used in the NNPAS 2011–12 can be found elsewhere [49]. For the present study, we used the data on physical activity, strength/toning activities, sedentary behaviour and selected sociodemographic and health-related variables.

#### Physical activity

Self-reported physical activity levels were assessed using the Active Australia Survey which has been previously validated among adults and older adults. The survey has been shown to have adequate reliability for classifying participants into insufficiently/sufficiently active groups (Cohen’s kappa = 0.50–0.52) (ref), and adequate validity when assessed against pedometer step counts (Spearman rho = 0.42–0.43) [50] and against accelerometers (Spearman rho = 0.52) [51]. Respondents reported the number of occasions (frequency) and estimated time spent (duration) in walking and other moderate-intensity activity (e.g. gentle swimming, social tennis doubles, golf), and vigorous physical activity (e.g. jogging, fast cycling, circuit training, competitive tennis) over the past week. It should be noted that these questions also covered participation in any moderate- or vigorous-intensity muscle-strengthening activity. The data were scored using established methods described in the guide for implementation of the Active Australia Survey [52]. The reported durations for each activity were summed to estimate the total time spent in MVPA. Based on the 2014 Australia’s Physical Activity & Sedentary Behaviour Guidelines for Adults [29], participants were dichotomised as either: (i) ‘meeting the MVPA guidelines’ (≥150 moderate-intensity minutes/week or ≥75 vigorous-intensity minutes/week or an equivalent combination of both), or (ii) ‘not meeting the MVPA guidelines’.

#### Muscle-strengthening activities

To assess participation in muscle-strengthening activities, respondents were asked, “Including any activities already mentioned, *in the last week did you do any strength or toning activities?”.* If they answered positively*,* they were further asked: *“How many times did you do any strength or toning activities in the last week?”.* It should be noted that these questions covered the muscle-strengthening activities that participants already mentioned in their responses to questions about MVPA, and potentially any other muscle-strengthening activities that are not of moderate or vigorous intensity. Similar questions have previously shown adequate reliability (Cohen’s kappa = 0.85–0.92) [53], and have been used in population studies in Australia [41] and the US [38, 45]. For strength and toning activities, data were missing for 1 participant. According to the Australian Physical Activity and Sedentary Behaviour Guidelines [29] and consistent with previous population studies [36, 38, 41, 43, 45], the sample was dichotomised as either; (i) ‘meeting the strength training guidelines’ (≥2 sessions/week), or (ii) ‘not meeting the strength training guidelines’ (<2 sessions/week).

#### Meeting the combined MVPA-strength training guidelines

Consistent with previous studies [36, 45, 54], based on the data about the participation in MVPA and muscle-strengthening activities, the sample was dichotomised as either (i) meeting the combined MVPA-strength training guidelines (≥150 MVPA minutes/week and ≥2 sessions/week of strength or toning activities) or (ii) not meeting the MVPA-strength training guidelines (<150 MVPA minutes/week or <2 sessions/week of muscle-strengthening activities).

#### Sedentary behaviour

Respondents reported their time spent in sedentary behaviour during the last week within the following contexts: (i) sitting at work (ii); sitting for transport (including waiting for transport); (iii) sitting or lying down to watch television or videos; (iv) sitting or lying down to play electronic games; (v) sitting or lying down to use a computer or the internet; (vi) sitting or lying down to use a phone (including text messages and talking); and (vii) sitting or lying down to do other social or leisure activities (such as at a barbecue, for meals, at a cinema, etc.). Similar questions have previously shown adequate reliability and validity in adults [55, 56]. Times spent sitting in these contexts were summed to calculate the total time spent in sedentary behaviour during the last week, and then divided by seven to report on minutes/day. Consistent with previous population studies [46], the sitting data were truncated at 960 min/day (16 h). A total of 45 out of 9435 cases (0.5 % of the total sample) were truncated for reporting sitting >960 min/day and 32 (0.3 % of the sample) were missing data on sedentary behaviour altogether.

At present there is no official agreement around the threshold at which sitting is considered detrimental for health. However, a recent meta-analysis showed that sitting for more than 8 h/day significantly increases the risk of all-cause mortality [57]. While further research is needed to validate this threshold, the potential for using 8 h/day as a measure of excessive engagement in sedentary behaviour was recently discussed by public health experts in the development of Australian sedentary behaviour guidelines [58, 59]. Therefore, in the present study, we considered ≥8 h/day (≥480 min/day) as a proxy measure of high engagement in sedentary behaviours, and classified participants as (i) ‘low-sedentary’ (<480 min/day) or (ii) ‘high-sedentary’ (≥480 min/day).

#### Participants at ‘high-risk’ - clustering of insufficient MVPA and strength training and high levels of sedentary behaviour

Respondents were classified in the ‘high-risk’ group if they: (i) did not meet the MVPA guidelines (<150 min/week), and (ii) did not meet the strength training guidelines (<2 sessions/week), and (iii) were in the ‘high-sedentary’ category (≥480 min/day).

#### Sociodemographic and lifestyle variables

Sociodemographic (sex, age, level of education) and health related variables (self-rated health and smoking status) were assessed during the interview using standard questions. Body Mass Index (BMI) was calculated based on objectively measured height and weight using standard methods and categorised into: <18.5 kg/m^2^ (underweight); from ≥18.5 kg/m^2^ to <25 kg/m^2^ (normal/acceptable weight range); from ≥25 kg/m^2^ to <30 kg/m^2^ (overweight); and ≥30 kg/m^2^ (obese). More detailed description of the sociodemographic and health-related data collected in NNPAS 2011–12 can be found elsewhere [49].

### Statistical analysis

Percentages and their 95 % confidence intervals (95 % CI) were calculated for the following categories: (i) meeting the MVPA guidelines; (ii) meeting the strength training guidelines; (iii) meeting the combined MVPA-strength training guidelines; (iv) ‘low-sedentary’ (<480 min/day); and (v) ‘high-risk’ (not meeting the MVPA guidelines/not meeting the strength training guidelines/‘high-sedentary’). Chi-squared tests (either based on testing an overall association or trend depending on the variable) were used to test the unadjusted differences between the prevalence rates by selected sociodemographic (sex, age, level of education) and health-related variables (self-rated health, BMI, smoking status).

A series of multiple logistic regression analyses was used to assess the associations between sociodemographic and health-related variables and: (i) meeting/not meeting MVPA guidelines; (ii) meeting/not meeting the strength training guidelines; (iii) meeting/not meeting the combined MVPA-strength training guidelines; (iv) being ‘low/high sedentary’; and (v) being/not being in the ‘high-risk’ group (not meeting the MVPA guidelines/not meeting the strength training guidelines/‘high-sedentary’). Each model included the following explanatory variables: sex (reference group [ref] = “male”); age (ref = “18–24 years”); education level (ref = “high”); self-reported general health status (ref = “excellent”); BMI (ref = “normal weight”); and smoking status (ref = “never smoked”). Adjusted odds ratios and their 95 % CIs were reported. Statistical analyses were conducted using IBM SPSS 22.0 statistical software (SPSS Inc. an IBM Company, Chicago, IL). For all statistical tests, a p-value of <0.05 was used to indicate statistical significance. All estimates were weighted using population weights provided by the ABS. Strata were not identifiable in the data due to confidentiality concerns, so the effect of the sample design on the accuracy of the estimates was accounted for by using the delete-a-group Jackknife method to calculate the standard error of the estimates. Replicate weights were provided by the ABS. More detailed information can be found on the ABS website [48].

## Results

Data were available for 9435 adults aged 18–85 years (response rate = 77.0 %). As shown in Table 1, when compared to current Australian population estimates [51], the proportions of the NNPAS 2011–12 sample was largely concordant across most sociodemographic variables. In the current study, a total of 54.1 % were female, 45.9 % were aged 18–44 years, 25.7 % had high education levels (degree or higher degree), 51.5 % reported either ‘excellent’ or ‘very good’ self-rated health, 34.3 % had normal BMI status and 48.5 % reported never smoking (Table 1).

**Table 1:** Prevalence of meeting moderate to vigorous-intensity physical activity (MVPA) guidelines^a^, strength training guidelines^b^, the combined MVPA-strength training guidelines^c^ and being ‘low-sedentary’^d^ – overall and by sociodemographic and health-related factors

	Current study^e^	Population-weighted estimates^f^	Met MVPA guideline^a^ (*n* = 9285)	Met strength training guideline^b^ (*n* = 9434)	Met both MVPA and strength guidelines^c ^ (*n* = 9284)	‘Low-sedentary’^d^ (<480 min/day) (*n* = 9435)
	n	N	% (95 % CI)^g^	% (95 % CI)^g^	% (95 % CI)^g^	% (95 % CI)^g^
Total	9435	17,042,208	52.6 (51.2–54.0)	18.6 (17.5–19.7)	15.0 (13.9–16.1)	78.9 (77.9–80.0)
Sex	n (%)^g^	n (%)^g^				
Male	4329 (45.9)	8,406,261 (49.3)	55.0 (52.8–57.3)	20.7 (19.2–22.2)	16.9 (15.4–18.4)	75.0 (73.2–76.8)
Female	5106 (54.1)	8,635,947 (50.7)	50.2 (48.2–52.2)	16.6 (14.9–18.1)	13.1 (11.6–14.7)	82.8 (81.5–84.0)
*p-value*			0.002*	<0.001*	<0.001*	<0.001*
Age						
18–24 years	780 (8.3)	2,233,305 (13.1)	62.2 (57.2–67.1)	29.2 (25.2–33.2)	25.3 (21.3–29.3)	80.9 (77.2–84.6)
25–34 years	1617 (17.1)	3,152,930 (18.5)	58.7 (54.7–62.6)	23.3 (19.9–26.6)	19.0 (15.6–22.3)	73.4 (70.7–76.1)
35–44 years	1843 (19.5)	3,147,104 (18.5)	53.4 (50.3–56.6)	18.5 (16.4–20.5)	15.3 (13.4–17.3)	76.8 (74.7–78.8)
45–54 years	1660 (17.6)	3,023,041 (17.7)	52.9 (49.3–56.4)	17.7 (14.9–20.5)	14.3 (11.8–16.8)	76.7 (73.9–79.5)
55–64 years	1432 (15.2)	2,565,640 (15.1)	48.7 (44.8–52.6)	14.2 (12.1–16.4)	11.1 (8.8–13.4)	78.7 (75.6–81.7)
65–74 years	1255 (13.3)	1,680,712 (9.9)	46.5 (42.4–50.7)	12.4 (10.2–14.6)	8.3 (6.3–10.4)	87.7 (85.2–90.1)
≥ 75 years	848 (9.0)	1,239,476 (7.3)	33.3 (29.4–37.1)	7.6 (5.4–9.7)	4.1 (2.3–5.8)	89.2 (86.4–92.0)
*p-value*			<0.001*	<0.001*	<0.001*	<0.001*
Education level						
Low (<Year 12 or no non-school qualification)	2579 (27.8)	4,252,855 (25.3)	40.2 (37.6–42.8)	11.5 (9.9–13.1)	8.5 (6.9–10.2)	84.0 (82.2–85.9)
Medium (Year 12, diploma or certificate)	4327 (46.5)	8,293,573 (49.4)	52.0 (50.0–54.0)	19.6 (17.8–21.3)	15.4 (13.8–17)	80.4 (78.6–82.1)
High (Degree or higher degree)	2385(25.7)	4,246,623 (25.3)	66.1 (64–68.1)	23.6 (21.2–26.0)	20.5 (18.2–22.8)	71.0 (68.8–73.3)
*p-value*			<0.001*	<0.001*	<0.001*	<0.001*
Self-rated health						
Excellent	1537 (16.3)	2,919,514 (17.1)	67.9 (65.4–70.4)	29.7 (26.1–33.3)	26.1 (22.5–29.7)	79.5 (76.5–82.4)
Very good	3223 (35.2)	6,172,432 (36.2)	57.4 (55.1–59.7)	21.5 (19.3–23.7)	17.9 (15.8–19.9)	79.8 (78.0–81.5)
Good	2965 (31.4)	5,316,252 (31.2)	47.9 (45.2–50.6)	14.1 (12.4–15.8)	10.4 (8.7–12)	78.8 (76.6–81.1)
Fair	1163 (12.3)	1,942,921 (11.4)	35.1 (30.3–39.9)	8.7 (6.4–11.1)	5.4 (3.6–7.1)	77.2 (73.9–80.5)
Poor	477 (4.7)	691,090 (4.1)	30.5 (24.1–36.9)	8.4 (4.5–12.3)	5.2 (2.1–8.3)	75.0 (67.8–82.2)
*p-value*			<0.001*	<0.001*	<0.001*	0.120
Body Mass Index (kg/m^2^)						
Underweight (<18.5)	121 (1.5)	280,115 (1.9)	54.4 (42.5–66.3)	12.6 (5.30–20.0)	8.6 (2.0–15.1)	84.6 (76.6–92.5)
Normal (18.5–25)	2735 (34.3)	5,262,928 (36.1)	59.4 (57.0–61.8)	24.2 (21.5–26.9)	20.5 (18.1–22.9)	80.3 (78.2–82.3)
Overweight (25–30)	2898 (36.4)	5,266,721 (36.1)	56.1 (53.6–58.6)	20.3 (18.2–22.4)	15.8 (13.8–17.9)	79.0 (77.1–80.9)
Obese (≥30)	2203 (27.7)	3,776,994 (25.9)	45.0 (42.4–47.6)	12.4 (10.7–14)	9.7 (8.2–11.3)	76.7 (74.2–79.1)
*p-value*			<0.001*	<0.001*	<0.001*	0.020*
Smoking status						
Never smoked	4573 (48.5)	8,776,577 (51.5)	53.7 (51.8–55.6)	19.0 (17.4–20.5)	15.5 (14.1–16.9)	79.5 (77.9–81.2)
Ex-smoker	3077 (32.6)	5,263,643 (30.9)	55.1 (53.1–57.0)	21.0 (19.0–23.0)	16.9 (15.1–18.8)	78.8 (76.8–80.9)
Current smoker	1785 (18.9)	3,001,988 (17.6)	45.0 (41.8–48.3)	13.4 (11.3–15.6)	10.1 (8.2–12.0)	77.4 (74.8–80.0)
*p-value*			<0.001*	0.003*	0.004*	0.140

^a^Prevalence of respondents who reported engaging in at least 150 min per week of moderate-intensity physical activity or 75 min of vigorous-intensity physical activity per week, or an equivalent combination of bothn

^b^Prevalence of respondents who reported participating in muscle-strengthening physical activity at least two times per week

^c^Meeting both the MVPA and strength training guidelines

^d^To be classified as ‘low-sedentary’, respondents had to report <480 min/day spent in sedentary behaviours

^e^National Nutrition and Physical Activity Survey (NNPAS) 2011–12 respondents

^f^Replicate weights generated from the Australian Bureau of Statistics - Australian Health Survey: Users' Guide, 2011–13 - 4363.0.55.001

^g^Percentages are weighted and are given relative to the total number within each sociodemographic and health-related variable

*p < 0.05: *X* ^*2*^ test for overall association for sex and trend for the other sociodemographic and health-related variables

**Table 2:** Adjusted odds ratios^a^ (OR), and their 95 % confidence intervals (95 % CI), of being classified as meeting moderate to vigorous-intensity physical activity (MVPA) guidelines^b^, meeting the strength training guidelines^c^, meeting the combined MVPA-strength training guidelines^d^ and being classified as ‘low-sedentary’^e^ – overall and by sociodemographic and health-related factors

	Met MVPA guideline^b^	Met strength training guideline^c^	Met both MVPA and strength guidelines^d^	‘Low-sedentary’^e^ (<480 min/day)
Explanatory variable	OR (95 % CI)	OR (95 % CI)	OR (95 % CI)	OR (95 % CI)
Gender (ref: male)				
Female	0.83 (0.71–0.95)	0.77 (0.65–0.91)	0.76 (0.61–0.93)	1.61 (1.39–1.87)
Age group (ref: 18–24 years)				
25–34 years	0.71 (0.51–0.98)	0.64 (0.49–0.85)	0.57 (0.41–0.80)	0.70 (0.54–0.91)
35–44 years	0.57 (0.43–0.75)	0.46 (0.36–0.60)	0.44 (0.32–0.60)	0.85 (0.63–1.14)
45–54 years	0.65 (0.49–0.85)	0.48 (0.35–0.67)	0.45 (0.32–0.64)	0.88 (0.65–1.20)
55–64 years	0.55 (0.41–0.75)	0.37 (0.29–0.49)	0.33 (0.23–0.45)	1.03 (0.78–1.36)
65–74 years	0.53 (0.39–0.71)	0.33 (0.24–0.46)	0.25 (0.18–0.37)	1.65 (1.14–2.37)
≥ 75 years	0.36 (0.25–0.52)	0.21 (0.14–0.32)	0.14 (0.08–0.24)	2.25 (1.37–3.70)
Education level (ref: high [Degree or higher degree])				
Medium (Year 12, diploma or certificate)	0.58 (0.51–0.66)	0.83 (0.67–1.03)	0.74 (0.60–0.92)	1.71 (1.46–2.01)
Low (<Year 12 and no non-school qualification)	0.49 (0.41–0.58)	0.73 (0.57–0.93)	0.70 (0.53–0.93)	2.04 (1.64–2.53)
Self-rated health (ref: excellent)				
Very good	0.69 (0.56–0.84)	0.65 (0.49–0.87)	0.63 (0.46–0.86)	0.99 (0.77–1.26)
Good	0.49 (0.40–0.60)	0.43 (0.33–0.55)	0.36 (0.28–0.47)	0.88 (0.68–1.16)
Fair	0.35 (0.26–0.46)	0.32 (0.23–0.46)	0.24 (0.16–0.36)	0.78 (0.58–1.05)
Poor	0.35 (0.24–0.50)	0.38 (0.20–0.73)	0.26 (0.11–0.61)	0.57 (0.35–0.92)
Body Mass Index (ref; normal [18.5–25])				
Underweight (<18.5)	0.81 (0.50–1.33)	0.39 (0.21–0.73)	0.31 (0.14–0.68)	1.33 (0.69–2.58)
Overweight (25–30)	1.01 (0.86–1.19)	0.94 (0.76–1.17)	0.89 (0.70–1.13)	0.96 (0.80–1.15)
Obese (≥30)	0.76 (0.64–0.89)	0.64 (0.50–0.81)	0.65 (0.50–0.85)	0.77 (0.63–0.93)
Smoking status (ref; never smoked)				
Ex-smoker	1.29 (1.14–1.47)	1.39 (1.16–1.68)	1.47 (1.20–1.80)	0.94 (0.76–1.15)
Current smoker	0.87 (0.73–1.05)	0.79 (0.63–0.99)	0.77 (0.59–0.99)	0.89 (0.73–1.08)

^a^Adjusted for all other explanatory variables in the model

^b^To meet the MVPA guideline respondents had to report engaging in at least 150 min per week of moderate-intensity physical activity or 75 min of vigorous-intensity physical activity per week, or an equivalent combination both

^c^To meet the strength training guideline, respondents had to report engaging in strength training at least two times per week

^d^Meeting both the MVPA and strength training guidelines

^e^To be classified as ‘low-sedentary’, respondents had to report <480 min/day spent in sedentary behaviours

For the total sample, the median time spent in MVPA was 150 min/week (95 % CI: 134–155, interquartile range [IQR]: 365). The majority of participants reported no participation in muscle-strengthening activities; hence the median number of sessions per week was 0. Among the 22.7 % of participants that did muscle-strengthening activities, the mean number of sessions per week was 3.66 (95 % CI: 3.34-23.86, SD = 3.01). The median time spent in sedentary behaviours was 308 min/day (95 % CI: 305–314, IQR: 253).

As shown in Table 1, 52.6 % (95 % CI: 51.2 %–54.0 %) of people met the MVPA guideline only, 18.6 % (95 % CI: 17.5 %–19.7 %) met the strength training guideline (ST) only and 15.0 % (95 % CI: 13.9 %–16.1 %) met both guidelines (MVPA-ST) (Table 1). Unadjusted analyses indicated significant differences between the proportions of participants meeting these guidelines across all sociodemographic and health-related variables (*p* < 0.001 for almost all comparisons) (Table 1).

Accordingly, as shown in Table 2, in the adjusted analysis, all the selected sociodemographic and health-related variables showed significant overall associations with meeting/not meeting MVPA guideline, the strength training guideline, and the combination of both guidelines (overall *p* < 0.05 for all explanatory variables). Specifically, females (OR = 0.76, 95 % CI: 0.61–0.93) were less likely to meet the combined MVPA-ST guidelines then males. When compared to adults aged 18–24 years, all other age groups were less likely to meet the combined MVPA-ST guidelines. The ORs ranged from 0.14 (95 % CI: 0.08–0.24) for the oldest age group to 0.57 (95 % CI: 0.41–0.80) for those at the age of 25–34 years. Compared to adults reporting ‘excellent’ self-rated health, all other groups were less likely to meet the combined MVPA-ST guidelines. The ORs ranged from 0.26 (95 % CI: 0.11–0.61) for those reporting ‘poor’ health to 0.63 (95 % CI: 0.46–0.86) for those reporting ‘very good’ health. When compared to those with high education, those with low (OR = 0.70, 95 % CI: 0.53–0.93) and medium education (OR = 0.74, 95 % CI: 0.60–0.92) were less likely to meet the combined MVPA-ST guidelines. Compared to those with normal BMI (18.5–25 kg/m^2^), those who were classified as obese (OR = 0.65, 95 % CI: 0.50–0.85) and underweight (OR = 0.31, 95 % CI: 0.14–0.68) were less likely to meet the combined MVPA-ST guidelines. Interestingly, when compared to those who never smoked, ex-smokers were more likely (OR =1.47, 95 % CI: 1.20–1.80), whilst current smokers were less likely (OR = 0.77, 95 % CI: 0.59–0.99) to meet the combined MVPA-ST guidelines.

A total of 78.9 % (95 % CI: 77.9 %–80.0 %) were classified as ‘low-sedentary’ (Table 1). In the adjusted analysis, overall associations with sedentary behaviour categories were significant for all the explanatory variables (overall *p* < 0.001 for all), except for smoking status (overall *p* = 0.49). Specifically, when compared to the reference groups, females (OR = 1.61, 95 % CI: 1.39–1.87), adults aged 65–74 years (OR = 1.65, 95 % CI: 1.14–2.37) and ≥75 years (OR = 2.25, 95 % CI: 1.37–3.70) and those with the low (OR = 2.04, 95 % CI: 1.64–2.53) or medium level of education (OR = 1.71, 95 % CI: 1.46–2.01) were more likely to be classified as ‘low-sedentary’. In contrast, when compared to the reference groups, obese individuals (OR = 0.77, 95 % CI: 0.63–0.93), those with poor self-related health (OR = 0.57, 95 % CI: 0.35–0.92) and those aged 25–34 years (OR = 0.70, 95 % CI: 0.54–0.91) were less likely to be classified as ‘low-sedentary’.

Overall, 8.9 % (95 % CI: 8.1–9.6) of the sample were categorised as ‘high-risk’ with regards to their physical activity and sedentary behaviour (Table 3). In the adjusted analysis, compared to adults reporting ‘excellent’ health, those in other self-rated health categories were more likely to be in the ‘high-risk’ behaviour group (Table 4). The ORs ranged from 1.65 (95 % CI: 1.12–2.44) for ‘very good’ health category to 4.60 (95 % CI: 2.89–7.33) for the ‘poor’ health category. When compared to the youngest age group, those aged 25–34 (OR = 1.91, 95 % CI: 1.27–2.87) and 35–44 years (OR = 2.09, 95 % CI: 1.27–3.44) were more likely to be in the ‘high-risk’ group. Current smokers (OR = 1.37, 95 % CI: 1.06–1.76) and obese individuals (OR = 1.44, 95 % CI: 1.04–1.98) were more likely to be classified in the ‘high-risk’ group than those who never smoked and those with normal weight, respectively.

**Table 3:** Percentage of Australian adults classified as ‘high-risk’^a^, based on reporting insufficient moderate to vigorous-intensity physical activity (MVPA), insufficient strength training participation, and high sedentary time - overall and by sociodemographic and health-related factors

	Current study^b^	Population-weighted estimates^c^	‘High-risk’^a^
	n	N	% (95 % CI)^e^
Total	867	1,500,637	8.9 (8.1–9.6)
Sex			
Male	450	805,012	9.6 (8.5–10.7)
Female	417	695,626	8.1 (7.0–9.2)
*p-value*			0.04*
Age			
18–24 years	53	136,364	6.1 (4.1–8.2)
25–34 years	146	287,331	9.1 (7.6–10.7)
35–44 years	172	325,118	10.4 (8.7–12.0)
45–54 years	165	271,123	9.0 (7.2–10.9)
55–64 years	158	263,115	10.3 (7.9–12.7)
65–74 years	108	119,006	7.1 (5.3–9.0)
≥ 75 years	65	98,581	8.1 (5.8–10.4)
*p-value*			0.49^e^
Education level			
Low (<Year 12 and no non-school qualification)	260	432,408	10.2 (8.8–11.7)
Medium (Year 12, diploma or certificate)	388	726,351	8.8 (7.5–10.1)
High (Degree or higher degree)	210	322,979	7.6 (6.3–8.9)
*p-value*			0.01*
Self-assessed health			
Excellent	1532	291,2947	4.9 (3.5–6.3)
Very good	3299	612,7479	7.4 (6.3–8.5)
Good	2950	5,297,893	9.4 (7.9–11.0)
Fair	1155	1,927,885	14.3 (11.4–17.2)
Poor	443	684,920	18.8 (13.8–23.8)
*p-value*			<0.001*
Body Mass Index (kg/m^2^)			
Underweight (<18.5)	121	280,115	7.2 (1.0–13.4)
Normal (18.5–25)	2723	5,228,776	7.1 (5.8–8.5)
Overweight (25–30)	2889	5,253,598	7.1 (5.70–8.50)
Obese (≥30)	2191	3,757,075	12.3 (10.3–14.3)
*p-value*			0.002*
Smoking status			
Never smoked	373	701,268	8.0 (7.0–9.1)
Ex-smoker	276	438,185	8.4 (7.1–9.6)
Current smoker	218	361,185	12.1 (10.2–14)
*p-value*			<0.001*

^a^‘High risk’ group defined as: insufficient MVPA (<150 min/week); AND insufficient strength training participation (<2 sessions/week); AND being classified as ‘high-sedentary’ (≥480 min/day)

^b^National Nutrition and Physical Activity Survey (NNPAS) 2011–12 respondents

^c^Replicate weights generated from the Australian Bureau of Statistics - Australian Health Survey: Users' Guide, 2011–13 - 4363.0.55.001

^d^Percentages are weighted and are given relative to the total number within each sociodemographic and health-related variable

^e^Whereas the trend test was not significant for age group, the *X* ^*2*^ test for overall association was 0.020

*p < 0.05: *X* ^*2*^ test for overall association for sex and trend for the other sociodemographic and health-related variables

**Table 4:** Adjusted odds ratios^a^ (OR), and their 95 % confidence intervals (95 % CI), of being classified as ‘high-risk’, based on reporting insufficient moderate to vigorous-intensity physical activity (MVPA), insufficient strength training participation, and high sedentary time

Explanatory variable	OR (95 % CI)
Gender (ref: male)	
Female	0.84 (0.70–1.01)
Age group (ref: 18–24 years)	
25–34 years	1.91 (1.27–2.87)
35–44 years	2.09 (1.27–3.44)
45–54 years	1.39 (0.91–2.12)
55–64 years	1.64 (0.98–2.75)
65–74 years	1.26 (0.80–1.98)
≥ 75 years	1.06 (0.58–1.96)
Education level (ref: high, completed year 12 or equivalent)	
Medium (completed year 10–11)	1.03 (0.77–1.39)
Low (completed year 9 or less)	1.07 (0.79–1.46)
Self-assessed health (ref: excellent)	
Very good	1.65 (1.12–2.44)
Good	2.10 (1.39–3.16)
Fair	2.96 (1.86–4.73)
Poor	4.60 (2.89–7.33)
Body Mass Index (ref; normal [18.5–25])	
Underweight (<18.5)	0.99 (0.39–2.51)
Overweight (25–30)	0.87 (0.66–1.16)
Obese (≥30)	1.44 (1.04–1.98)
Smoking status (ref; never smoked)	
Ex-smoker	0.94 (0.75–1.18)
Current smoker	1.37 (1.06–1.76)

^a^Adjusted for all other explanatory variables in the model

^b^‘High risk’ group defined as: insufficient MVPA (<150 min/week); AND insufficient strength training participation (<2 sessions/week); AND being classified as ‘high-sedentary’ (≥480 min/day)

## Discussion

This paper is the first to concurrently establish the prevalence and correlates of MVPA, strength training and sedentary behaviour among a national-representative sample of Australian adults.

The key finding of this study is that the vast majority (85 %) of Australian adults did not meet the full physical activity guidelines that incorporate both MVPA and strength training. Previous studies assessing the prevalence of physical inactivity among Australian adults have been solely based on MVPA levels, and show that 40 %–50 % of Australians are insufficiently active for health [5, 35, 60]. However, our results suggest that when combining strength training and MVPA levels, the prevalence of physical inactivity far exceeds previous estimates. In fact, our findings suggest that estimating population adherence to the physical activity recommendations using only MVPA data may be largely misleading and may not reveal the true extent of the problem of inactivity.

When compared to the findings from U.S. population-based studies [36, 44, 45], fewer Australian adults met both the MVPA and strength training guidelines (15.0 % vs. 18.2 %–20.6 %). Our prevalence estimate for meeting the strength training guidelines is slightly higher than in previous Australian studies. Among ~5, 800 participants from the Australian Diabetes, Obesity and Lifestyle (AusDiab) study [42], ~5, 700 from a sample of older Australian adults (≥65 years) [45], and ~1, 200 adults from regional Australia [41], the prevalence range was 9.4 %–15.5 % . However, when compared to data from previous U.S. and UK studies, our findings suggest that strength training participation among Australian adults is somewhat lower (18.6 % vs. 21.9 %–31.7 %) [36–40]. Furthermore, when compared to the most recent physical activity prevalence data from the WHO Global Health Observatory [35], the proportions of Australians in our sample meeting the MVPA guidelines is somewhat similar.

The importance of physical inactivity from a clinical perspective was highlighted in a recent report released by the Australian Institute of Health and Welfare [61]. In that report, physical inactivity was identified as the most prevalent risk factor for cardiovascular disease (57 % [note: based on insufficient MVPA levels only]), followed by high cholesterol (32.8 %) and high blood pressure (30 %). Given the substantial health benefits associated with regular participation in both MVPA and strength training, the low prevalence of Australian adults meeting these guidelines is of serious concern for public health. Comprehensive approaches are needed to promote and support both aspects of physical activity concurrently at the population level.

The sociodemographic correlates of strength training and MVPA observed in this study are consistent with existing data. Previous research has shown that older age, lower education levels and having poor health are associated with a lower prevalence of strength training [62], MVPA [33] and combined strength training and MVPA [37]. Our data underscore the importance of targeting these population groups in health promotion strategies. Particularly concerning was the finding that over 80 % of Australians do not engage in sufficient strength training. Research suggests that, independent of MVPA, strength training has beneficial outcomes which are important for health and wellbeing, such as prevention and treatment of diabetes [12, 63] and cognitive decline [10] and improvements and maintenance of skeletal muscle mass/strength [10], bone mineral density [64] and physical functioning [65]. More research is now needed to examine the key factors influencing strength training participation [66]. However, when contrasted the decades of research examining the correlates and predictors of leisure-time physical activity [33, 67, 68], comparatively little is known about the key factors influencing strength training.

This study examined only a small number of potential correlates of MVPA and muscle-strengthening activities. Future studies should move beyond this and examine other potential socio-demographic, lifestyle, psychological (e.g. motivation, intentions), social (e.g. social support, perceived social norms) and environmental factors (e.g. access to facilities, affordability) influencing MVPA and strength training participation in the Australian adult population. Additionally, given that strength training often requires equipment and specific knowledge in exercise instruction, future research may be needed to evaluate the effectiveness of incentives, such as subsidising equipment (e.g. resistance bands, dumbbells) and fitness/health club memberships, to increase muscle-strengthening activity participation rates.

The percentage of participants classified as ‘low-sedentary’ (<480 min/day) was somewhat lower than in a large Australian study among ~6700 women [22] and in a study from Japan among ~83,000 middle-aged and older adults [69] (78.9 % vs. 83.5 % and 92.7 %, respectively). These slight variations are likely to be explained by differences in samples and the use of diverse sedentary behaviour assessment tools. . Being classified as ‘high-sedentary’ was associated with overweight/obesity, higher education and poorer self-rated health. These findings are somewhat similar with research on the correlates of high volumes of siting, which show a relationship between high sitting volumes and poor health status, high BMI and high education levels [46, 70]. The inverse associations between age and sitting time are consistent with previous large-scale studies using similar self-report measures [46, 47]. Furthermore, there are a number of other potential correlates of sedentary behaviour [71]; however their analysis was beyond the scope of this paper.

To our knowledge, this is the first study to determine the proportion of the population at potentially high risk, based on the clustering of ‘not meeting the MVPA guidelines’, ‘not meeting the strength training guidelines’ and being classified as ‘high-sedentary’. Assuming that there may be cumulative health risks associated with insufficient physical activity and excessive sedentary behaviour, this population group are of a particular public health concern. The fact that there are up to 18.8 % of people at the ‘high-risk’ in different population subgroups, should motivate public health stakeholders to put even greater efforts in targeting prevention. The odds of being classified as ‘high-risk’ were 4.6 times higher among adults who reported ‘poor’ health when compared to those who reported ‘excellent’ health. Those classified as obese had ~44 % higher odds to be in the ‘high-risk’ group than individuals with normal BMI. These findings are somewhat consistent with MVPA/strength training correlates research [33, 45], and further underscore the need to target such groups in health promotion strategies. Further studies using this clustering approach of combining those with insufficient MVPA/strength training levels and high levels of sedentary behaviour are needed to compare and contrast our findings.

Strengths of this study include the involvement of a large national-representative sample of Australian adults [48]. Furthermore, the current study enabled assessment of physical activity and sedentary behaviour across a variety of sociodemographic and health-related variables. A further strength was the use of standardised MVPA, strength training and sedentary behaviour assessment instruments, which allowed for comparisons with other studies.

Limitations of the study were the use of self-report measures of MVPA, strength training and sedentary behaviour, which may have resulted in recall bias [72]. To improve the validity of estimates, future studies might consider using accelerometers and inclinometers to assess time spent in MVPA and sedentary behaviours/sitting, and time-use diaries to assess strength training participation. Nevertheless, for public health surveillance, standardised self-report instruments seems to be the method of choice for assessing the physical activity and sedentary behaviour levels [73]. Furthermore, no data was available on breaks in sitting time. Some of the participants classified as ‘high sedentary’ might have reduced health risks of prolonged sitting by taking regular breaks in sedentary behaviour [74]. The frequency of breaks in sitting time among Australian adults remains to be explored in future national surveys. A further limitation to this study was that, given its cross-sectional design, the direction of causality could not have been determined. For example, it may be that obesity lead to increased sitting time [75], but it is also possible that the direction of causality was opposite [76]. Furthermore, this study investigated only a small number of selected sociodemographic and lifestyle variables related to MVPA, muscle-strengthening activity and sedentary behaviour. Future studies are needed to identify and describe other potential correlates.

## Conclusions

This study showed that the vast majority of Australian adults do not meet the full PA recommendation that incorporates both MVPA and strength training. In particular, our findings showing the low levels of strength training among Australian adults warrant attention. While strength training is an important component of physical activity-related health, it has practically been ignored by public health approaches to chronic disease prevention. In addition to the continual population monitoring of strength training and MVPA levels, public health interventions should target subgroups at the highest risk of low participation levels in these physical activity-related behaviours (e.g. adults aged 55+ years, females and those with low-education levels).

Furthermore, it seems that interventions to reduce sitting time should target males, younger age groups, those with high level of education, obese individuals and those with poor self-rated health. Finally, multifaceted interventions may be needed for those with poorer self-rated health, obese individuals, those aged 25–44, and current smokers, as they are at the highest risk of high sedentary behaviour combined with insufficient MVPA and muscle-strengthening activity.
